# What influences the outcome of active disinvestment processes in healthcare? A qualitative interview study on five recent cases of active disinvestment

**DOI:** 10.1186/s12913-021-06298-3

**Published:** 2021-04-01

**Authors:** Adriënne H. Rotteveel, Mattijs S. Lambooij, Joline J. A. van de Rijt, Job van Exel, Karel G. M. Moons, G. Ardine de Wit

**Affiliations:** 1 Centre for Nutrition, Prevention and Health Services, National Institute for Public Health and the Environment (RIVM), PO Box 1, 3720 BA Bilthoven, the Netherlands; 2 Erasmus School of Health Policy & Management, Erasmus University Rotterdam, PO Box 1738, 3000 DR Rotterdam, the Netherlands; 3 Julius Center for Health Sciences and Primary Care, University Medical Center Utrecht, Utrecht University, PO Box 85500, 3508 GA Utrecht, the Netherlands; 4 Erasmus School of Economics, Erasmus University Rotterdam, PO Box 1738, 3000 DR Rotterdam, the Netherlands

**Keywords:** Health technology reassessment, Policy process, Agenda-setting, Policy formulation, Decision-making, Determinants, Qualitative research

## Abstract

**Background:**

Recent attempts of active disinvestment (i.e. withdrawal of reimbursement by means of a policy decision) of reimbursed healthcare interventions in the Netherlands have differed in their outcome: some attempts were successful, with interventions actually being disinvested. Other attempts were terminated at some point, implying unsuccessful disinvestment. This study aimed to obtain insight into recent active disinvestment processes, and to explore what aspects affect their outcome.

**Methods:**

Semi-structured interviews were conducted from January to December 2018 with stakeholders (e.g. patients, policymakers, physicians) who were involved in the policy process of five cases for which the full or partial withdrawal of reimbursement was considered in the Netherlands between 2007 and 2017: benzodiazepines, medication for Fabry disease, quit smoking programme, psychoanalytic therapy and maternity care assistance. These cases covered both interventions that were eventually disinvested and interventions for which reimbursement was maintained after consideration. Interviews were transcribed verbatim, double coded and analyzed using thematic analysis.

**Results:**

The 37 interviews showed that support for disinvestment from stakeholders, especially from healthcare providers and policymakers, strongly affected the outcome of the disinvestment process. Furthermore, the institutional role of stakeholders as legitimized by the Dutch health insurance system, their financial interests in maintaining or discontinuing reimbursement, and the possibility to relieve the consequences of disinvestment for current patients affected the outcome of the disinvestment process as well. A poor organization of patient groups may make it difficult for patients to exert pressure, which may contribute to successful disinvestment. No evidence was found of a consistent role of the formal Dutch package criteria (i.e. effectiveness, cost-effectiveness, necessity and feasibility) in active disinvestment processes.

**Conclusions:**

Contextual factors as well as the possibility to relieve the consequences of disinvestment for current patients are important determinants of the outcome of active disinvestment processes. These results provide insight into active disinvestment processes and their determinants, and provide guidance to policymakers for a potentially more successful approach for future active disinvestment processes.

**Supplementary Information:**

The online version contains supplementary material available at 10.1186/s12913-021-06298-3.

## Background

Over the last decades, OECD countries have spent an increasing proportion of their gross domestic product (GDP) on healthcare [1–3]. It is expected that healthcare expenditures will keep growing faster than the GDP due to demographic changes and the introduction of technological innovations, increasing the pressure on public financing [3, 4]. To limit this increase in healthcare spending, governments worldwide have introduced cost-saving policy initiatives, such as lean thinking and retracting inefficient processes [5]. Furthermore, proposals for disinvestment (i.e. withdrawal, restriction, retraction or replacement [6, 7] of healthcare interventions and services have been made [5, 8, 9]. Reasons for disinvestment may be, among others, ineffectiveness, unsafety or a lack of value for money [10, 11]. Furthermore, disinvestment can either be passive (i.e. not dependent upon direct policy intervention) or active (i.e. requiring direct policy intervention, such as a reimbursement decision) [12].

In the Netherlands, adults are obliged to buy individual private health insurance. Although this health insurance is provided on a competitive health insurance market, the coverage (i.e. the basic benefits package) is similar for everyone [13]. Most types of curative care are automatically included in the basic benefits package, except for outpatient pharmaceutical care and expensive inpatient pharmaceutical care. For these two types of care, assessment and appraisal by the National Healthcare Institute (*Zorginstituut Nederland*; ZiNL) and a subsequent positive decision by the Minister of Health, Welfare and Sports (hereafter referred to as ‘the Minister’) are a prerequisite for health insurance coverage. ZiNL uses four package criteria for its formal assessment, i.e. effectiveness, cost-effectiveness (i.e. do the effects outweigh the costs), necessity (i.e. do disease severity and the costs per patient justify coverage), and feasibility (i.e. is coverage feasible), with effectiveness being a knockout criterion [14–16].

For interventions that are covered by the basic benefits package, it is assumed that if they do not meet the package criteria anymore (e.g. because of new evidence or because of new alternatives becoming available), they will no longer be delivered by healthcare providers (passive disinvestment), so that explicit exclusion of these interventions (active disinvestment) is not necessary [14]. However, in recent years, the Minister has taken several decisions to exclude specific interventions from the basic benefits package, indicating that the assumption that existing interventions not meeting the package criteria anymore will automatically be (passively) disinvested may not always hold [17, 18]. Hence, active disinvestment may be needed.

Active disinvestment may also be necessary in the case of conditional reimbursement (also called: coverage with evidence development). In the Netherlands, it is possible to conditionally reimburse promising healthcare interventions that do not (yet) meet the package criteria in a special arrangement that is targeted at collecting evidence on the effectiveness and cost-effectiveness of these interventions. In such an arrangement, healthcare interventions will be temporarily reimbursed, provided that additional research will be conducted. After a predetermined time, usually 4 years, the reimbursement of the intervention will be reassessed using the findings from this additional research, which may result in a disinvestment decision [19, 20].

In the international literature, active disinvestment has been described as a very difficult process [21], hampered by contextual aspects such as political barriers [22, 23], a lack of dedicated resources [22], and disinvestment being considered counter-cultural [24]. Furthermore, it has been suggested that, in order to obtain more insight into this difficult process, qualitative studies are needed [23]. Active disinvestment processes in the Netherlands have clearly differed in their outcome. Some attempts at disinvestment resulted in actual disinvestment. Other attempts were terminated at some point, implying unsuccessful disinvestment. Currently, not much is known about active disinvestment processes in the Netherlands and what aspects (package criteria versus other considerations and contextual aspects) determine their outcome. Therefore, here, we aimed to obtain more insight into active disinvestment processes and to explore what aspects determine their outcome. We used a qualitative approach consisting of semi-structured interviews, which enabled us to obtain in-depth insight in the factors influencing the outcome of active disinvestment processes.

## Methods

This study focused on policy processes in which the active disinvestment of healthcare interventions or services, which have previously been (conditionally) reimbursed to patients through the basic health insurance, was considered. Active disinvestment includes the full withdrawal, restriction, retraction or substitution of the reimbursement of healthcare interventions or services by means of a policy decision [6, 12]. In policy making, several stages can be distinguished (i.e. the policy cycle): 1) agenda-setting, 2) policy formulation, 3) decision-making, 4) policy implementation and 5) policy evaluation [25]. As the aim of this study was to assess the outcome of active disinvestment processes (i.e. what decision was made with regards to disinvestment), this study mainly focused on the agenda-setting, policy formulation, and decision-making stages of active disinvestment processes.

### Theoretical framework

We have used the three essential elements in policy making - actors, ideas and structures [25] - to select distinct cases (see Selection of cases), to inform data collection (i.e. to inform the interview guide, see Data collection) and to structure data analysis (i.e. to structure the coding tree, see Data analysis). *Actors* are people or organizations who either 1) try to influence policy to advance their interests or 2) are being influenced by policy. The *ideas* actors have, can range from particular points of view to a widely held, sustained belief system. Arguments are expressions of these ideas. The *structures* in which actors operate are arenas in which policy processes take place and that affect the role that actors and their ideas can play in policy-making [25]. In addition, we have used the model of the organization of the Dutch Healthcare system [26] to guide data collection (i.e. identify categories of actors that may have been involved in the cases studies) and structure data analysis (i.e. to determine actor codes).

### Selection of cases

This study focused on five distinct cases to study active disinvestment processes. Possible cases were interventions for which disinvestment was formally and actively considered in the period 2007–2017. This includes interventions that have eventually been actively disinvested, either fully or partially, and interventions for which reimbursement has been maintained. Through screening of policy documents from ZINL and the Ministry of Health, Welfare and Sports (hereafter referred to as ‘the Ministry’) that were found on the websites of ZiNL and the Ministry using specific search terms (e.g. stopping reimbursement, disinvestment), 34 possible cases for which active disinvestment was considered, were identified (see Additional file A). From these 34 possible cases, 5 were selected that were expected to be distinct in 1) the actors, ideas (i.e. reasons for considering disinvestment) or structures involved, and 2) the outcome of the disinvestment process (fully or partially disinvested or reimbursement maintained). Cases were (year of decision in brackets): Benzodiazepines (that are mainly used for anxiety and sleeping disorders; 2007, 2008), Medication for Fabry disease (2012), Quit smoking program (2009, 2011, 2012), Psychoanalytic therapy (2010), and Maternity care assistance (in Dutch: Kraamzorg; 2015). See Additional file B for a description of the selected cases.

### Data collection

Semi-structured interviews were conducted with actors who were involved in the disinvestment process for the selected cases. Involved actors were identified from policy documents, through media coverage of the case and through interviews with other actors, and approached through e-mail. New respondents were included for each case until 1) no new information regarding the disinvestment process of the case emerged from the interviews (i.e. informational redundancy/data saturation was reached for the case [27]), 2) all eligible actors for the case were interviewed (i.e. no other actors were involved), or 3) there were no eligible actors left who were willing to participate. The perspectives of the different actor groups were complementary, with the aim to get a complete picture of the disinvestment process of the case. We did not aim to reach saturation with regards to the perspectives of the different actor groups on disinvestment.

The interview topic list was developed for this study and covered actors, ideas, and structures involved in the agenda-setting, policy formulation, and decision-making stage of the disinvestment process. Besides, if relevant, we asked participants to reflect on differences and similarities between the disinvestment process for the case discussed and other reimbursement processes (both investment and disinvestment) they were involved in (if any). The semi-structured interview guide is included in Additional file C. Interviews were audio-recorded and transcribed verbatim.

#### Ethical considerations

Written informed consent was obtained at the start of the interview. Respondents were explained that only the research team would have access to the audio recordings and transcripts of their interviews, and that they would handle this information confidentially. Furthermore, respondents were explained that, with regards to publications on this study, no names of persons and/or organizations (apart from ZiNL and the Ministry), nor any specific information which would reveal the identity of respondents would be reported. Quotes that were used in this paper were approved by respondents, without them having the possibility to change anything regarding the findings. This research project has been assessed by the Centre for Clinical Expertise (CCE) at the RIVM. The CCE concluded that the research project is exempted from further review by a medical ethics committee as it does not fulfill the specific conditions as stated in the Dutch Medical Research Involving Human Subjects Act (WMO; *Wet Medisch-wetenschappelijk Onderzoek met mensen)*.

### Data analysis

Interview transcripts were analyzed using thematic content analysis [28]. Transcripts were coded by AR, using a combination of deductive and inductive coding. Text segments from the transcripts were coded using the essential elements in policy making (actors, ideas and structures; see Theoretical framework). The part of the coding tree for the essential element ‘ideas’ was derived from the Dutch package criteria and the review of Kleinhout-Vliek et al. [14, 29, 30]. The complete coding tree is displayed in Additional file D. Coding was conducted using MAXQDA 2019 [31].

A subset of 15 interviews was also coded by JvdR to assess the reliability of data analysis [32]. This subset was purposively selected to include three interviews on each case and to include interviews with different types of respondents. Within this subset of 15 interviews, there was sufficient consensus between both coders on the text fragments coded and the codes used to code these fragments. Therefore, double coding of the remaining interviews was deemed unnecessary (i.e. remaining interviews were only coded by AR).

To facilitate the analysis, coded text segments were aggregated in three matrices (one for actors, one for ideas and one for structures), with the rows in these matrices displaying the codes and the columns displaying the cases, arranged from most disinvested (i.e. reimbursement fully stopped) to least disinvested (i.e. decision to continue reimbursement). The cells in the matrices contained a short summary of the information in the text segments coded under the respective code for each case. The matrices were inspected by AR, ML and GdW for recurring themes and trends on the disinvestment process itself and aspects determining the outcome of this process. To this end, the authors focused on the differences and commonalities between the studied cases, especially between the cases with similar or very different outcomes. The final main themes were determined through discussion between AR, ML and GdW, focusing on aspects that appeared to have had the largest (or a surprisingly small) impact on the outcome of the disinvestment process in the studied cases. The final themes were checked for plausibility and approved by the second coder based on his/her knowledge of the data from coding a subset of the interviews.

## Results

In total, 37 interviews with 37 respondents and an average duration of 53 min were conducted between January and December 2018. Of the 37 interviews, 32 were held with one respondent, four with two respondents, and one with four respondents. Of the 37 respondents, 5 were interviewed on multiple cases (counted as separate interviews). Most interviews were administered by one interviewer (AR). In four interviews, a second interviewer joined (GdW or N.J.E. van Vooren) because of the number of respondents taking part in the interviews. A timeline displaying the reimbursement status and the policy reports published on the case was brought to the interviews to stimulate remembrance and to support the conversation (see Additional file E). Table 1 shows the number of interviews that was conducted per case and per respondent group. In four out of five cases, either data saturation was reached and/or all eligible actors were interviewed. In the cases where saturation was reached, no new information on the disinvestment process of the case was obtained from the last interviews conducted. In the quit smoking program case, not all eligible actors were interviewed as we did not succeed to include a politician who was involved.

**Table 1:** Number of interviews conducted per case and per respondent group

Case	Respondent group						Total number per case
	Patient representatives^**a**^	Healthcare providers^**b**^	Health insurers	National Healthcare Institute (ZINL)	Ministry of health, welfare and sports	Other	
Benzodiazepines	1	2	1	2	2	1^c^	9
Medication for Fabry disease	1	1	1	1	1	1^d^	6
Quit smoking program	1	0^e^	1	1	1	3^f^	7
Psychoanalytic therapy	1	3^g^	1	1	NA^h^	1^c^	7
Maternity care assistance	1	3^i^	1	1	2	0	8
**Total over all cases**	5	9	5	6	6	6	37

^a^ This respondent group includes patients or professionals who acted as patient representatives at the time of the disinvestment process

^b^ The respondent group healthcare providers includes both (leading) individual healthcare providers as well as healthcare provider organizations (e.g. physician associations)

^c^ i.e. knowledge institute

^d^ i.e. pharmaceutical company

^e^ In this case, several interviewees are from interest groups that represent both patients and healthcare providers as well (i.e. different actor groups are united in one organization). Therefore, the healthcare provider perspective has been covered in the other interviews

^f^ i.e. interest groups + advisor of interest groups

^g^ In this case, in the first interview with a healthcare provider, we identified two even more involved healthcare providers. Therefore, in total, three interviews with healthcare providers were necessary for this case

^h^ Disinvestment decisions based on (lack of evidence of) effectiveness can be taken by the National Healthcare Institute and do not need involvement by the Ministry. For this reason, the Ministry was not involved in the disinvestment decision regarding psychoanalytic therapy, and was, hence, not interviewed

^i^ Several healthcare provider organizations are involved in maternity care assistance. Therefore, we needed three interviews with healthcare providers to interview all relevant healthcare provider organizations

The remaining part of the results section is structured around the five main aspects influencing the outcome of disinvestment processes, as identified in our analysis. Here, the differences and similarities of these themes between cases are discussed. Additional file F contains a description of these themes for each case, separately. From this point forward, the cases are indicated with an alphabetical code instead of the case name to protect the anonymity of the respondents.

### Theme 1: support for active disinvestment and pressure exerted

In our interviews, respondents described that the degree of support for disinvestment from actors, including the public, was important for the outcome of the disinvestment process. However, the importance of support differed between actors and cases (see Additional file F and the sections below). Respondents from all cases described that the degree of support for disinvestment was related to actors’ views on the case (i.e. health problem and intervention/service concerned), and in some cases to the attention for and framing of the case in the media. Respondents described that for them, as well as for other actors, the degree of support influenced the actions undertaken, and, subsequently, the amount of pressure exerted. From a comparison of the interview data from the studied cases, we found that the pressure exerted had a large effect on the outcome of the disinvestment process: limited pressure was exerted in the cases that were actually disinvested, while much pressure was exerted in the cases for which reimbursement was maintained. In the following quote, a healthcare provider describes the effect of societal pressure on the outcome of the disinvestment process.

**Healthcare provider, case D, reimbursement maintained:**

*“Yes, I believe that the social pressure, especially all media attention, including emotional arguments, helped a lot to reach this conclusion [not to stop reimbursement] in the end.”*

#### Support from healthcare providers

Respondents described that especially the support from healthcare providers was important for the outcome of the disinvestment process (i.e. has an important effect on the decision that policymakers make):

**Interest group, case C, first disinvested, later reimbursed again:**

*“Respondent: And [the minister] actually had less of a say in that, I think. I think that the social pressure, particularly on this item from the healthcare sector, which she ultimately needs much more, also for the rest of her policy, that it is higher.*

*Interviewer: In that respect, surely the lobby and the calls from the healthcare sector were very important in this?*

*Respondent: To reverse it [the decision], yes, definitely.”*

By comparing the interview data from the studied cases, we found that the cases where healthcare providers successfully exerted pressure against disinvestment were not-disinvested, while cases where healthcare providers did not exert pressure or were unsuccessful in exerting pressure were disinvested. According to respondents, whether healthcare providers were successful in exerting pressure depended on 1) their willingness to exert pressure, subject to a) their level of support for disinvestment of the case, b) whether they feel it fits their role (e.g. whether they feel they have an advisory role which does not comply with exerting pressure) and c) their (financial) interests, and 2) the possibility they had to exert pressure, subject to a) their awareness on how to exert pressure and b) the opportunities provided to them to do so. In the first quote below, a healthcare provider from case B explains that they did not exert pressure because of their belief that disinvestment would improve healthcare delivery (i.e. other healthcare providers would better follow guidelines), suggesting their support for disinvestment. In the second quote a healthcare provider from case A describes his/her difficulty in convincing other actors to join forces (i.e. being successful) in exerting pressure.

**Healthcare provider, case B, partially disinvested:**

*“And, and so in that, up to that point we were like yes, if it actually helps to have it paid for [by patients], [ …]* *and if that helps them [other health care providers] to follow our guideline better, and to give less [of the treatment], yes, then it is fine of course.”*

**Healthcare provider, case A, fully disinvested:**

“*Interviewer: Was there any party that you managed to get on board?*

*Respondent: No, not really. [...] I can tell it like it is. At least people paid lip service to this whole story. People understood and supported the arguments. But to say that [name] who was then president of [organization] was like ‘will you state once and for all that [case] is indispensable in the Netherlands and at any cost... ‘, [name] never did that.”*

#### Support from governmental institutions

From our comparison of the interviews, we found that the opportunity provided to actors, including healthcare providers, to exert pressure seemed to depend for a large part on the opportunity actors were given by governmental institutions, who are in charge of disinvestment processes, to be involved in the policy process. We observed large differences between cases in the opportunity respondents were provided to be involved in the policy process, which seemed to depend on the support from governmental institutions for the case at hand. This is illustrated by the quotes below describing the opportunity respondents felt they were provided to be involved in case E, where policymakers described they were not in favor of disinvestment, and case A, where respondents suggested that policymakers were in favor of disinvestment.

**Healthcare provider, case E, reimbursement maintained:**

*“Interviewer: But they [ZiNL] were willing to listen to you in any case?*

*Respondent: Definitely.*

*Interviewer: And did they also accept them, um, did they agree with the arguments you put forward?*

*Respondent: Yes, they did. But I can still remember that they, and it’s of course very good that they always wanted to investigate for themselves, were like “okay, we can’t accept everything just like that”.”*

**Patient organization, case A, fully disinvested:**

*“And well, they [ZiNL] are always going to mention in their advice that they have spoken with – for example, right – in our case [organization name] with so and so. Yes, I can put that into perspective. Those five minutes that it took for us to quickly hand over a box of signatures, well, yes, even if it was a bit longer, that’s not really a conversation, is it? There’s no way you have really heard us.”*

From our comparison of the interview data between cases, we found that the support from governmental institutions also seemed to affect the use of the formal assessment framework in the cases studied. We observed differences between cases in respondents’ descriptions of the criteria that were used in the disinvestment process, how these criteria were interpreted, and how these criteria were weighted against one another. For instance, as is illustrated by the quotes below, in case A, where respondents suggested policymakers were in favor of disinvestment, the focus was mainly on whether the treatment was evidence-based, while in case E, where policymakers described they were not in favor of disinvestment, more considerations than strictly related to the four package criteria were taken into account in the policy process.

**Healthcare provider, case A, fully disinvested:**

*“The motive was: we only reimburse things that are evidence-based. Well, a lot of what’s in the package just isn’t evidence-based so that’s kind of a weird reasoning.”*

**Policymakers from ZiNL, case E, reimbursement maintained:**

*“You could say it was a very heterogeneous set of effect measures and because of that diversity and heterogeneity, well, it is sort of, it is kind of a multi-factor analysis, right, of all those factors. Are they pointing in the same direction? Do they all contribute to the goal that you want to achieve with [the case]? Well, and that answer was yes in the end.”*

#### Support from patients

Respondents from cases A and B, which were actually disinvested, described that patients in these cases were very vulnerable due to their disease and the societal problems associated with it. Because of this, according to respondents, patients were poorly organized, which made it difficult to get a large group of patients to exert pressure against disinvestment, as is illustrated by the quote below. This lack of pressure exerted may have facilitated disinvestment in these cases.

**Patient organization, case A, fully disinvested:**

*“Yes, for most people it is a taboo, a long-term [treatment] like that. Especially when you’re still in the middle of it. So, it was difficult to reach the patients. [ …] It was almost impossible, it was a real taboo, there is so much going on when you get such a long-term [treatment].”*

### Theme 2: compassion for current users

Respondents described that the degree of support from actors for disinvestment was affected by whether measures could be taken to ease the effect of disinvestment for patients currently using the intervention at hand. This is illustrated by the quotes from policymakers below in which they describe that they consider disinvestment much more difficult than not to start reimbursement in the first place, especially if disinvestment results in current patients having to stop their treatment or switch to another treatment.

**Civil servant from the Ministry, case D, reimbursement maintained:**

*“But you know, you are dealing with people who have been under treatment for years, um, and who are confronted with a new reality overnight. So, that also played a role, also for the Ministry. Like “yeah, you know, what is wisdom, you can’t stop people’s treatment just like that”.”*

**Policymaker ZiNL, case A, fully disinvested:**

*“And we said, “People should be allowed to finish their treatment.“ That was actually, there was some discussion about that. What were the arguments? I don’t know. I think, I don’t know how explicit that was but, yes, a reliable government, you started [the treatment] after all, yes.”*

Respondents described that if measures could be taken to relieve the effect of disinvestment on current patients, such as stopping reimbursement only for new patients or only restricting reimbursement, they considered disinvestment much more acceptable. This indicates that the possibility to take such mitigating measures impacts the support for disinvestment from actors and, subsequently, the outcome of the disinvestment process.

### Theme 3: role in the health insurance system

From the interviews, we observed that the role appointed to actors by the Dutch health insurance system also affected the outcome of the disinvestment process. In the Dutch health insurance system, two groups of policy makers advise the Minister on reimbursement (policy formulation): ZiNL is involved with reimbursement aspects with regards to content, while policy makers of the Ministry itself both focus at the content and context of reimbursement. Once the Minister decides on reimbursement, health insurers subsequently implement these reimbursement decisions, and patients/healthcare providers are both consulted in reimbursement decisions and in the implementation of these decisions. Every actor can be involved in agenda-setting, although policymakers often have the most distinct role in this phase. In our interviews, respondents described that for the cases studied, actors acted in accordance with the role they have in the system. They described that actors tended to stay away from pursuing actions that they considered to be beyond their formal role. For instance, in the quote below, a health insurer explains that they generally refrained from trying to influence policy formulation and decision-making on reimbursement, as they only consider the implementation of reimbursement decisions to be part of their role.

**Health insurer, case D, reimbursement maintained:**

*“Actually, over the years, we’ve had less and less of an opinion about this sort of thing. Because we feel, you know, everyone has their part to play in the system.”*

Although this finding was most distinct for health insurers, other respondents also regularly discussed their tendency to stick to their formal role. As was described in the interviews, if actors felt that exerting pressure was beyond their formal role, they generally refrained from this. Hence, the formal role of actors affected the pressure that was exerted by these actors in the cases studied and, subsequently, the outcome of disinvestment processes.

### Theme 4: financial interest in disinvestment

From the interviews, we observed that the actions actors took and, subsequently, the outcome of the disinvestment process were also affected by the financial interest of actors in the outcome of the disinvestment process. Respondents described that actors who had a financial interest in maintaining reimbursement were more likely to take action and exert pressure against disinvestment, than actors who did not have a financial interest in maintaining reimbursement, as is demonstrated by the quote below:

**Healthcare provider, case B, partially disinvested:**

*“No, because all those [case intervention] were no longer, there wasn’t a patent on them anymore, so they were... It wasn’t in those manufacturers interest anymore to interfere. Otherwise it would probably not have been possible [to stop reimbursement]. Then they would have pulled out all the stops, to undo that.”*

### Theme 5: role of the formal package criteria

From our interviews, no consistent pattern of the influence of the formal Dutch package criteria (i.e. effectiveness, cost-effectiveness, necessity and feasibility) on the outcome of the disinvestment process was observed. This finding is illustrated by the fact that our study included two cases (i.e. case A and D) that, based on what respondents shared with us during the interviews, seemed to score similarly on the formal package criteria, while having a very different outcome (fully disinvested versus maintained), a different outcome that cannot be explained by the cases scoring differently on any other criterion as well.

## Discussion

To our knowledge, this is the first study to describe the agenda-setting, policy-formulation and decision-making stages of the active disinvestment process, and to explore aspects influencing its outcome. By using a qualitative approach, we were able to study the reasoning behind the choices made in the active disinvestment process of the five cases studied in-depth and to collect information that has not been included in the formal policy reports written about these cases. Moreover, through interviewing different actors, we were able to look at the disinvestment process from different perspectives, in addition to the formal perspective of policymakers obtained from policy reports.

Our findings indicate that it is more likely for active disinvestment processes to result in a disinvestment decision if 1) there is sufficient support for disinvestment from actors, especially from policymakers and healthcare providers, 2) it is possible to ease the effect of disinvestment for patients currently using the intervention, 3) actors do not have a financial interest in maintaining reimbursement, and 4) actors are not inclined to exert pressure against disinvestment beyond their formal role. Furthermore, we found that the effect of the formal Dutch package criteria on the outcome of the disinvestment process differed between cases.

### Comparison with previous studies

Our finding that support, especially from healthcare providers and policymakers, is an important determinant of the outcome of the disinvestment process also has been described by previous studies in which support, especially from clinical leaders, but from the general public as well, was found to be essential for the success of the disinvestment process [6, 33, 34]. Furthermore, a lack of support from decision-makers has been described as a barrier to the implementation of disinvestment [17]. However, it has also been described in previous studies that obtaining support for disinvestment is very difficult as stakeholders often lack the will to support disinvestment because of limited perceived value of disinvestment, which may take some time to realize, and their resistance to immediately “losing” therapy options [12, 35]. Hence, to make actors, especially healthcare providers and policymakers, aware of the value of disinvestment (e.g. quality improvements, reducing displacement) may be essential in order to obtain support and, subsequently, to ensure that a decision to disinvest an intervention is taken and implemented. A review from 2008 focusing on reimbursement decisions for new medicines also found healthcare providers and administrators (i.e. policymakers) to be most influential in reimbursement decisions [36], indicating that this aspect may be similar in investment and in disinvestment decisions.

In our study, we found that limiting the effect of disinvestment on current patients increased support for disinvestment and, subsequently, facilitated actual disinvestment, as actors were reluctant to disrupt treatment of current patients. This has also been found in two previous studies in which citizens and policymakers expressed a reluctance to disrupt treatments because of the negative effect this may have on current patients, e.g. anxiety because of losing the current treatment option and side-effects of stopping/changing treatments [6, 37]. Hence, when considering the disinvestment of healthcare interventions, it is important to consider if and how the treatment of current patients could be (partially) continued. This consideration may be less relevant in the context of investment as, in the investment context, patients are not yet receiving the appraised intervention, and hence, no treatment will be disrupted if a decision not to reimburse the intervention is taken. This indicates that different considerations may be relevant in the context of disinvestment compared to the context of investment.

In this study, we found that actors’ financial interests influence the outcome of the active disinvestment process through the actions actors undertake. Similar findings have been described by previous studies on the disinvestment/de-implementation of low value care. For instance, van Egmond et al. found that financial incentives influence the de-implementation of low-value care by healthcare providers [38]. Furthermore, Haas et al. and Robinson et al. describe that there is a lack of financial incentives for healthcare providers to engage in disinvestment [24, 39]. Further research is needed to determine how changing financial incentives may facilitate disinvestment. To our knowledge, our finding that actors’ inclination to exert pressure because of the role appointed to them by the health insurance system influences the outcome of active disinvestment processes has not been found by previous studies on the disinvestment of healthcare interventions.

In the Netherlands, a formal assessment framework is in place for the appraisal of new healthcare interventions for reimbursement. However, in this study we found that the formal criteria from this assessment framework for new additions to the basic benefits package (i.e. effectiveness, cost-effectiveness, necessity and feasibility) have been inconsistently applied in disinvestment decisions. This finding may be problematic as a structured, evidence-based process has been described in a review from 2015 as a facilitator for (the implementation of) disinvestment [17]. Furthermore, consistency in the rationale underlying decisions has been described as an important requirement for decision-making to be considered fair [40]. Therefore, it is important for policymakers to ensure that the criteria underlying disinvestment decisions are consistently applied. Such a consistent assessment framework may also facilitate the selection of the potentially most successful (i.e. most likely to result in actual disinvestment) cases for active disinvestment processes.

Previous studies have described that, although broad stakeholder engagement is considered essential for the success of disinvestment, it is considered very difficult to sufficiently organize this [17, 22, 24]. Although this aspect has not been specifically addressed in the current study, we did find that the possibility for stakeholders to be involved in the disinvestment process differed between cases, depending on the support from policymakers for disinvestment. Furthermore, we found that the engagement of patients was difficult, especially in cases where the patient group was in a vulnerable situation and, because of this, poorly organized.

### Strengths and limitations

In this study, we have used a qualitative approach which enabled us to obtain in-depth insight into the factors that influence disinvestment processes and their outcomes. Furthermore, by interviewing many different actors who have perceived the disinvestment process from different perspectives, we were able to obtain a comprehensive view of the disinvestment processes studied. The perspectives of all the different actors were of added value and treated equally, complementing each other in the reconstruction of the disinvestment processes. Moreover, as a sample (40%) of the interview transcripts was double coded, and the matrices were checked for recurring themes by three researchers, we ensured the reliability of data analysis [32]. In addition, as the disinvestment process of the studied cases took place several years ago, it may have been perceived as a closed chapter by respondents, making elaborating on the cases less sensitive and reflecting on it easier.

However, our study also has some limitations. Firstly, we have selected 5 cases from a list of 34 identified cases. Although we aimed to select 5 distinctive cases from this list, we may have missed aspects determining the outcome of the disinvestment process that may have been more pronounced in the non-selected cases. Furthermore, as we identified the 34 possible cases from policy documents, we may have introduced bias, as we could only select cases that made it to the stage of a policy document. Because of this, we may have missed cases for which the disinvestment process was terminated at a very early stage, before any policy document was written. However, even though it is clear that some information may have been omitted due to case selection, we did include a distinct selection of cases to maximize the transferability of our findings to other cases.

Secondly, although we tried our best to have all the relevant topics discussed during the interviews, it may be that respondents (intentionally or unintentionally) left some relevant information out. Especially one of the five cases studied (i.e. case D) still appeared to be sensitive to respondents, indicating that it may be more prone to information omission. We tried to overcome this limitation by including multiple respondents for each case. No conflicting information was obtained from the interviews with the different respondents, indicating that there are no clear signs of information omission. Furthermore, the older cases may have also been more prone to information omission because respondents may have had trouble remembering the relevant information. We tried to overcome this by showing respondents a timeline displaying policy reports and the reimbursement status to stimulate remembrance. In the interviews, this timeline appeared to be helpful for respondents in remembering the disinvestment process.

Thirdly, in the quit smoking program case we were not able to interview all eligible actors because we did not succeed in including a politician who was involved in this case. As politicians played a major role in this case, it would have been informative to interview a politician. However, as the political process was extensively discussed in the remaining interviews on the case, we expect the information omission by not including politicians to be limited.

Finally, this study has been conducted in a Dutch setting. As disinvestment processes are often context-specific [21], one should take the differences between healthcare systems and other contextual factors into account when considering the implications of this study for other healthcare systems. For instance, in other healthcare systems, the roles may be differently divided between the different actors than is the case in the Dutch healthcare system. Moreover, actors may have a different tendency to stick to these roles. This may affect the importance of this aspect for the outcome of disinvestment processes in other healthcare systems. Therefore, to find out which aspects determine the outcome of disinvestment processes in other contexts, future research may consider conducting a similar assessment as has been conducted in this study. Note, however, that conducting multiple interviews per case is dependent on the willingness of key actors to share their experience. In our current study, almost all approached actors were willing to participate. We believe that this large willingness to participate may have been caused by the fact that the disinvestment processes of the cases took place several years ago. Therefore, to increase the willingness of key actors to participate in future studies, we recommend to select cases that may be perceived as a closed chapter, but that are recent enough for actors to sufficiently remember the disinvestment process.

### Implications

The results of this study may have implications for future active disinvestment processes. If policymakers are interested in selecting candidate interventions for which the disinvestment process is more likely to result in a disinvestment decision, they are recommended to select interventions for which there is support for disinvestment from actors, especially from healthcare providers. Furthermore, interventions for which support can be easily obtained, for instance because of the possibility to ease the consequences of disinvestment for current patients, are suitable candidate interventions as well. Moreover, also interventions for which actors do not have (strong) financial interests in maintaining reimbursement are suitable candidates for disinvestment.

After having selected candidate interventions for disinvestment, policymakers are recommended to increase support for disinvestment to facilitate the disinvestment process. As support from healthcare providers appeared to be important, policymakers are recommended to start with increasing support among this actor group. To this end, healthcare providers who are not aware of all the potential merits of disinvestment should be made aware of these merits in addition to the possible disadvantages they may already be aware of (i.e. losing therapy options for new patients and disrupting treatment of current patients). In order to make these healthcare providers aware of the merits, the scientific literature suggests that the emphasis of disinvestment processes should be on safety, and quality improvements such as avoiding side effects of ineffective care, instead of cost savings as this makes disinvestment more acceptable to healthcare providers [18, 41]. Furthermore, it is important to make these healthcare providers aware of the opportunity costs of providing the intervention that will be disinvested. For instance, healthcare providers should be explained that, by disinvesting the intervention, their time and efforts can be devoted to interventions that generate more health outcomes. Moreover, healthcare providers should be made aware of the displacement taking place in the healthcare system because of the reimbursement and usage of interventions that are unsafe or not (cost-)effective. In addition, as easing the consequences of disinvestment on current patients appeared to increase support, it is recommended to explore the opportunities to do so for candidate interventions for disinvestment. In order to facilitate disinvestment, policymakers are also recommended to explore the financial interests that actors have with regards to disinvesting or maintaining reimbursement of the intervention and the mechanisms underlying these financial interests. By being aware of this, policymakers can better anticipate on the pressure that will be exerted by actors because of these financial interests.

It is important for policymakers to be aware of the fact that different considerations may be relevant in the disinvestment context compared to the investment context. For instance, the reluctance to disrupt treatment for current patients may be highly relevant with regards to disinvestment decisions, while not being relevant in the investment context. As consistency and transparency in the rationale underlying disinvestment decisions is an important requirement for the policy process to be considered fair [40], we recommend policymakers to develop, consistently apply and transparently communicate on an assessment framework for disinvestment decisions, taking into account considerations that are specifically relevant in the disinvestment context.

#### Implications for conditional reimbursement

Conditional reimbursement, or coverage with evidence development is the reimbursement of (new) healthcare interventions under a specific condition, often the requirement of collecting real-world (cost-)effectiveness data. It has been proposed as a policy-tool to delay the final reimbursement decision, decreasing the uncertainty on the (cost-)effectiveness and/or budget-impact of new interventions, while making these interventions available to patients at an early stage [42, 43]. However, conditional reimbursement has also been described as creating a wedge effect, i.e. giving suppliers a “foot in the door” while creating challenges for decision-makers when the reversal of (conditional) reimbursement is warranted [12]. As the intervention has already been provided to patients, reversal of reimbursement implies the withdrawal of treatment, which has been described in the scientific literature as far less acceptable to actors than withholding treatment [42, 44]. However, the specific nature of conditional reimbursement processes, provides policymakers with opportunities to address the aspects influencing the likelihood of an active disinvestment process to result in a disinvestment decision, as identified in our study. For instance, conditional reimbursement has the advantage that support for disinvestment can be ensured before starting reimbursement. Therefore, to ensure support, policymakers are recommended to make agreements with actors, before starting reimbursement, on the criteria the intervention should meet to maintain reimbursement after the evaluation period. Furthermore, we recommend policymakers to change the default outcome of conditional reimbursement from ‘reimbursement is maintained if the intervention meets the criteria’ to ‘reimbursement is reversed, unless the intervention meets the criteria’. Although this difference may seem trivial, it has shown to be important psychologically [45, 46]. Subsequently, policymakers are recommended to make sure that actors understand and agree that if the intervention does not meet the agreed upon criteria at the end of the reimbursement period, it will be disinvested. In this way, policymakers ensure that actors know that care is only provided under the precondition that it can be withdrawn later on, which makes withdrawing treatment similar to withholding treatment from a psychological point of view [42, 44].

## Conclusions

In conclusion, this study indicates that contextual aspects such as support, institutional structures and financial interests are important determinants of the outcome of active disinvestment processes. Furthermore, the possibility to relieve the consequences of disinvestment for current patients appeared to be important to increase the acceptability of disinvestment. These results provide researchers and policymakers with insight into disinvestment processes in the Netherlands. Furthermore, the results can be used by policymakers in the selection of future candidate interventions for disinvestment and in future disinvestment processes to increase the likelihood of the disinvestment process to result in an actual disinvestment decision. Moreover, the results can be used by policymakers to prevent some of the challenges inherent in reversing reimbursement after a period of conditional reimbursement.

## Supplementary Information

**Additional file 1.**

## Data Availability

The interviews analyzed during the current study are not publicly available due to concerns for respondents’ privacy. However, a summary of the interview data on each theme for each case is included in additional file F.

## Abbreviations

CCE: Centre for Clinical Expertise
GDP: Gross Domestic Product
OECD: Organisation for Economic Cooperation and Development
ZINL: Dutch National Healthcare Institute
