# Supplementary material for: What influences the outcome of active disinvestment processes in healthcare? A qualitative interview study on five recent cases of active disinvestment
Source: BMC Health Serv Res. 2021 Apr 1;21:298. doi: 10.1186/s12913-021-06298-3 (PMC8017606; doi:10.1186/s12913-021-06298-3)
Supplement: Supplementary file 1 — Additional file 1. [file 12913_2021_6298_MOESM1_ESM.docx]

# Additional files paper disinvestment processes

## Additional file A: Identified cases, categorized (selected cases are marked yellow)

| **Type of disinvestment** | **Main reason for disinvestment (according to policy documents):** | | | |
| --- | --- | --- | --- | --- |
|  | **Effectiveness** | **Cost-effectiveness / budget cuts** | **Necessity** | **Feasibility** |
| **Full disinvestment** | NESS handmaster (2007) | Quit smoking interventions (2012) | | Diane-35 pill (2014) |
|  | Allergy-free covers (2009) | Medication Fabry disease (2013) | Stand-up-chair (2009) | Non-complicated extractions (2011) |
|  | Acetylcysteine (2010) | Medication Pompe disease (2013) | Rollator, crutches, walker (2013) | Circumcision (2013) |
|  | Psychoanalytic therapy (2010) | Ranibizumab (2015) | Contraceptives (2011) | |
|  | Intravesical sodium chondroitin sulphate/ hyaluronic acid (2014) | Statins (2009) | Helmet therapy (2013) |  |
|  | Renal denervation (2017) |  | Second opinion (2016) |  |
|  | Radiofrequent denervation (2016) |  | Fax machines for auditory impaired (2009) |  |
|  | Contralum ultra (2016) |  | Maternity care assistance (2016) |  |
|  | Paracetamol-codeine (2013) |  | Intracavernous fentolamine /papaverine (2009) |  |
| **Restriction** |  | Antacida (2012) | Dentist (2011) | Benzodiazepines (2009) |
|  |  | Diet advise (2012) |  |  |
|  |  | Fertility treatment (2013) |  |  |
|  |  | Anti-depressants (2011) |  |  |
|  |  | Physiotherapy (2011 & 2012) |  |  |
| **Retraction** |  |  | Incontinence products (2012) |  |
|  |  | Curative mental healthcare (2012) |  |  |
| **Replacement** |  |  |  |  |

## Additional file B: Description of the selected cases

**Benzodiazepines**

Benzodiazepines are sedative medicines that are mainly used for sleep and anxiety disorders. The policy process for the disinvestment decision took place in 2007 and 2008. Starting January 2009, benzodiazepines are no longer reimbursed through the basic benefit package. Exceptions were, however, made for five indications: epilepsy, therapy-resistant anxiety disorder, multiple psychiatric problems, palliative sedation, and therapy-resistant muscle spasms because of neurological disorders (the latter concerns only diazepam, no other benzodiazepines).

**Medication for Fabry disease**

Fabry disease is a hereditary, chronic and slowly progressive disease. In early disease stages, patients mainly suffer from pain in hands and feet, and a high body temperature. In later stages, patients experience symptoms of kidney failure, heart disorder and stroke. The disinvestment process concerned two orphan medicines, Agalsidase alpha and Agalsidase beta. The aim of these medicines is to decrease disease symptoms and to slow the progression of the disease. The medicines have been conditionally reimbursed, and were reassessed in 2012. In the reassessment, it was decided to keep reimbursing the medicines after (successful) price negotiations.

**Quit smoking programme**

The quit smoking programme includes two treatment options: 1) behavioral counselling, and 2) behavioral counselling combined with medicines. Starting January 2011, the quit smoking programme was included in the basic benefit package. A year later, starting January 2012, the quit smoking programme was disinvested from the basic benefit package. One year after that, starting January 2013, the quit smoking programme was, again, included in the basic benefit package and has remained reimbursed since. For our study, we mainly focused on the disinvestment in 2012 and the re-introduction in 2013.

**Psychoanalytic therapy**

The reimbursement decision, actually, concerned two therapies within the psychoanalysis field: psychoanalytic therapy and long-term psychoanalytic psychotherapy (LPPT). Psychoanalytic therapy is mainly focused on opening up subconscious aspects of dysfunctional behavior and dysfunctional experiences. It consists of four to five consultations a week, for five to ten years, in which the patient is laying down on a sofa. The psychoanalyst is sitting out of sight and mainly listens and reacts to what patients tell him/her. LPPT is a less intensive form of psychoanalytic therapy. It consists of one or two consultations a week, for one to two years. Instead of lying on the sofa with the analyst out of sight, the patient is sitting down, facing the analyst. The disinvestment process mainly took place in 2009 and 2010. Starting 2010, psychoanalytic therapy is no longer reimbursed through the basic benefit package. LPPT was not disinvested.

**Maternity care assistance**

Maternity care assistance (in Dutch: kraamzorg) is postpartum care for newborns and their mothers. The maternity care assistant provides care at home^[[1]](#footnote-1)^ in the first days (usually 8 days) after birth, under the supervision of a midwife. The main aim of maternity care assistance is to monitor and support a quick recovery of the mother and the appropriate development of the newborn. The main tasks of the maternity care assistant are nursing care for the newborn and the mother, providing health-education, signaling health problems, (breast) feeding support, and support with basic household tasks (to promote a hygienic environment). In 2012, in a Dutch tv-show, the minister of Health, Welfare and Sports called the public for suggestions to save healthcare costs. Some reactions to this call concerned stopping the reimbursement of maternity care assistance. Therefore, the minister asked the National Healthcare Institute (ZINL) for an advice regarding the reimbursement of maternity care assistance. In 2015, ZINL advised the minister to keep reimbursing maternity care assistance and this advice was followed by the minister.

## Additional file C: Semi-structured interview guide

### Introduction

As was described in the information letter, this study of the RIVM concerns disinvestment. Disinvestment is 1) the complete withdrawal of reimbursement, 2) the restriction of reimbursement, 3) the retraction of budget from reimbursement or 4) the replacement of healthcare interventions by alternatives.

In this study, the focus is on the disinvestment of healthcare interventions from the basic benefit package of health insurance. It concerns care which has been reimbursed to patients before. (Hence, it also concerns care which has erroneously been delivered at the expense of the basic benefit package because a lack of effectiveness had not yet been determined.)

To obtain more insight into active disinvestment processes and to explore what aspects determine their outcome, we interview stakeholders who were involved in disinvestment processes. In these interviews, we focus on a number of cases of disinvestment. These are both cases in which a disinvestment decision has been taken, as well as cases that only have gone through some stages in the disinvestment process, because of which no disinvestment decision has been taken. For instance, this concerns cases on which only an advice of the National Healthcare Institute has been written.

This interview concerns [case]. We approached you for an interview as you have been involved in the disinvestment process of this case.

To be able to analyze the interviews in an objective way, I ask you for your permission to record the interviews using an audio recorder. This recording will be used to write down (transcribe) the interview for analysis. We will send you the transcribed interview, so that you have the opportunity to check it for inaccuracies. We will handle both the audio recording as the transcribed interview confidentially. Only the research team will have access to this information.

The results of this study will be published. We may use one of your quotes in this publication to substantiate the results. We will, however, ensure that both the results and the quotes will not contain information that may reveal your identity.

Is everything clear? Do you agree with:

- Participation in this study?
- The audio recording and writing down of the interview?
- The inclusion of the results and possible quotes in a publication?

Do you have any questions before we will start with the interview?

[ask respondent to fill-out and sign informed consent form, start audio recording]

### Interview

**Introduction**

| **Topic** | **Questions** |
| --- | --- |
| General | As discussed before, this interview concerns [case]. |
|  | Later on I will pursue this case further. However, as a starting question: could you give a short summary of the case and your involvement in it? |

**Agenda-setting**

| **Topic** | **Possible questions^^[[2]](#footnote-2)^^** |
| --- | --- |
| General | What was the reason to consider whether the reimbursement of [case] should be changed? |
| Actors | Which parties/stakeholders were involved? |
| Ideas | What was your idea/opinion on the case? |
|  | Which ideas/opinions did other parties/stakeholders have on the case? |
|  | Which arguments were raised? |
| Structures | What was your role in this case? Formally? In practice? |
|  | What role did other stakeholder have? Formally? In practice? |
|  | To what extent did you have an influence on whether reimbursement would be changed? |
|  | To what extent did other stakeholders have an influence on whether reimbursement would be changed? |
|  | What was the role of the different arguments? |
|  | To what extent were the arguments a decisive factor in the disinvestment decision? |

**Policy formulation**

| **Topic** | **Possible questions** |
| --- | --- |
| General | Which options for reimbursement have been discussed? |
|  | Have other policy options been discussed in which reimbursement would not be changed? |
|  | How were these policy options identified? |
|  | How were these policy options assessed? |
| Actors | Which parties/stakeholders were involved in the identification and the assessment of the different policy options? |
| Ideas | Which ideas/opinions did these parties/stakeholders have on the case? |
|  | What was your idea/opinion on the different policy options? |
|  | Which ideas/opinions did other parties/stakeholders have on the policy options? |
|  | What arguments were raised by the parties/stakeholders? |
| Structures | What was your role? Formally? In practice? |
|  | What role did other stakeholder have? Formally? In practice? |
|  | To what extent did you have an influence on the identification and assessment of policy options? |
|  | To what extent did other parties/stakeholders have an influence on the identification and assessment of policy options? |
|  | What was the role of the different arguments? |
|  | To what extent were the arguments a decisive factor in the identification and assessment of policy options? |

**Decision-making**

| **Topic** | **Possible questions** |
| --- | --- |
| General | What was the final decision? |
|  | How has this decision been taken? |
| Actors | Which parties/stakeholders were involved? |
| Ideas | Which ideas/opinions did these parties/stakeholders have on the case? |
|  | What arguments were raised by the parties/stakeholders? |
| Structures | What was your role? Formally? In practice? |
|  | What role did other stakeholder have? Formally? In practice? |
|  | To what extent did you have an influence on the final decision? |
|  | To what extent did other parties/stakeholders have an influence on the final decision? |
|  | What was the role of the different arguments? |
|  | To what extent were the arguments a decisive factor for the final decision? |

**Actors who were involved, but have not yet been discussed/actors who were not involved**

| **Topic** | **Possible questions** |
| --- | --- |
| Involved, but not yet discussed | Were there any other parties/stakeholders that were involved in the disinvestment process on the case, but have not yet been discussed? |
|  | What were their ideas/arguments raised by them? |
|  | What was their role in the process? |
| Not involved | Were there any parties/stakeholders who were not involved in the disinvestment process on this case, but who are affected by the outcome of this process? |
|  | What were their ideas/arguments raised by them? |
|  | Why were they not involved in the process? |

**Comparison with other cases (if applicable)**

| **Topic** | **Possible questions** |
| --- | --- |
| Disinvestment cases | From your perspective, did the disinvestment process of [the case discussed] differ from disinvestment processes of other cases?   - Comparable cases? - Cases in which a decision was/was not taken? |
| Investment decisions | From your perspective, did the disinvestment process of [the case discussed] differ from the assessment of new treatments for the inclusion in the basic benefit package?   - Actors - Ideas - Structures |

### Closing

- Are there other things you would like to discuss on this case? Are there any relevant issues that have not yet been discussed?
- Do you have any written/internal documents on this case I may have a glance over?
- Are there other things you would like to discuss on disinvestment processes?
- Are there other stakeholders you think I should interview on this case? If so, who could we approach for this?
- Thank respondent and close off

## Additional file D: coding tree

| **Conceptual framework** | **Main codes** | **Subcodes** | | |
| --- | --- | --- | --- | --- |
| Essential elements | Actors | Ministry of Health, Welfare and Sports |  |  |
|  |  | Dutch Healthcare Institute (ZiNL) |  |  |
|  |  | Patient(s) (organizations) |  |  |
|  |  | Health care provider (organizations) |  |  |
|  |  | Manufacturers |  |  |
|  |  | Health insurers |  |  |
|  |  | Media |  |  |
|  |  | Knowledge institutes |  |  |
|  |  | *Politicians/ members of parliament*^1^ |  |  |
|  |  | *Minister/ secretary of state of Health, Welfare and Sports* |  |  |
|  |  | *Health Council* |  |  |
|  |  | *ZonMW (The Netherlands Organisation for Health Research and Development)* |  |  |
|  |  | *SER (Social and economic council)* |  |  |
|  |  | *Health funds* |  |  |
|  |  | *Partnership quit smoking* |  |  |
|  |  | *Stivoro (organisation against smoking)* |  |  |
|  |  | *Tobacco industry/lobby* |  |  |
|  |  | Other |  |  |
|  | Ideas | Effectiveness | Common practice |  |
|  |  |  | Efficacy |  |
|  |  |  | Effectiveness |  |
|  |  |  | Variability |  |
|  |  |  | Strength of evidence | *Level of evidence* |
|  |  |  |  | *Amount of research* |
|  |  |  |  | *No evidence is not evidence of ineffectiveness* |
|  |  |  |  | *Methodological quality* |
|  |  |  | Statistical significance |  |
|  |  |  | Clinical relevance |  |
|  |  |  | Patient relevance |  |
|  |  |  | Safety/adverse events |  |
|  |  |  | *Limitations in doing research* |  |
|  |  |  | *Necessity for further research* |  |
|  |  |  | *Mechanism of action* |  |
|  |  | Cost-effectiveness | ICER vs reference value |  |
|  |  |  | *Costs (per unit of the intervention)* |  |
|  |  |  | *QOL/QALYs* |  |
|  |  |  | Uncertainty |  |
|  |  |  | *Strength of evidence* | Methodological quality |
|  |  |  | Necessity of further research |  |
|  |  | Necessity | Necessity to insure | Definition of illness |
|  |  |  |  | *Definition of treatment/ what is covered by the basic benefit package* |
|  |  |  |  | Individual Cost |
|  |  |  |  | Individual responsibility |
|  |  |  |  | Moral Hazard |
|  |  |  |  | Range of Normality |
|  |  |  |  | Societal responsibility |
|  |  |  | Medical Necessity | Medical Necessity |
|  |  |  |  | Morbidity/Severity |
|  |  |  |  | Need |
|  |  |  |  | Rule of Rescue |
|  |  |  | Other necessity aspects | Dignity |
|  |  |  |  | Equity/fairness/justice |
|  |  |  |  | Human Right |
|  |  |  |  | (No) Alternative |
|  |  |  |  | Patient-diagnosis |
|  |  |  |  | Similar Treatments |
|  |  |  |  | Number of Patients |
|  |  |  |  | Societal impact |
|  |  |  |  | Societal functioning |
|  |  |  |  | Vulnerability/compassion |
|  |  |  |  | *Has been used to treat patients/reimbursed before* |
|  |  | Feasibility | Support |  |
|  |  |  | Organization of care |  |
|  |  |  | Indication and administration |  |
|  |  |  | Financing |  |
|  |  |  | Legal and ethical |  |
|  |  |  | Consultation and anticipation |  |
|  |  |  | Commencing date |  |
|  |  |  | Consequences for healthcare consumption | *Substitution* |
|  |  |  | Budget impact | Societal |
|  |  |  |  | Health care budget |
|  |  | Other | *Considered/fair process* | *Agenda-setting (sitting duck, arbitrariness)* |
|  |  |  |  | *Policy-development (criteria/ process)* |
|  |  |  |  | *Decision-making* |
|  |  |  |  | *Implementation* |
|  |  |  | *Healthcare delivery* | *Tailored care* |
|  |  |  |  | *Quality of care* |
|  |  |  | *Undesirable use* |  |
|  |  |  | *Autonomy healthcare providers* |  |
|  |  |  | *Trustworthy government* |  |
|  |  |  | *Disappearance/ erodation of a field* |  |
|  |  |  | *Reimbursement status gives a signal* |  |
|  |  |  | *Values* | *Solidarity* |
|  |  |  |  | *Accessibility* |
|  |  |  | *Prevention (value of)* | *Signaling health problems* |
|  |  |  | *Emotional arguments* | *You want to be able to do something for your patient as a temporary relieve* |
|  |  |  | *Financial arguments* | *Need to cut healthcare budget* |
|  |  |  |  | *Profit/returns* |
|  |  |  |  | *Having budget/financing* |
|  |  |  |  | *Sunk costs* |
|  |  |  |  | *Defend price setting* |
|  | Structures | Social/cultural | *Societal vision/ image/ idea* |  |
|  |  |  | *Clinical vision/ image/ idea* |  |
|  |  | Economic |  |  |
|  |  | Political | *Feeling of urgency* |  |
|  |  |  | *Momentum* |  |
|  |  |  | *Compromise* |  |
|  |  |  | *Societal pressure* |  |
|  |  |  | *Political pressure* |  |
|  |  |  | *Loss of face/ reputation* |  |
|  |  |  | *Political vision* |  |
|  |  |  | *Polarised debate* |  |
|  |  | Institutional/ regulatory | *Roles/ tasks organizations* |  |
|  |  |  | *Drug regulations/ approval* |  |
|  |  |  | *Withdrawing government* |  |
|  |  | *Historical* |  |  |
|  |  | *Financial* |  |  |
|  |  | *Other policies* |  |  |
|  |  | *Other reimbursement decisions* |  |  |
|  |  | *Investment vs disinvestment* |  |  |
|  |  | *Other reports* |  |  |
|  |  | Other |  |  |
| Policy cycle | Agenda-setting |  |  |  |
|  | Policy development | *Reimbursement options* |  |  |
|  | Decision-making |  |  |  |
|  | *Implementation* | *Side effects process* |  |  |
|  |  | *Uncertainty about reimbursement* |  |  |
| ^1^Codes in italics have been inductively added to the coding tree during the coding process | | | | |

## Additional file E: Timelines brought to the interviews


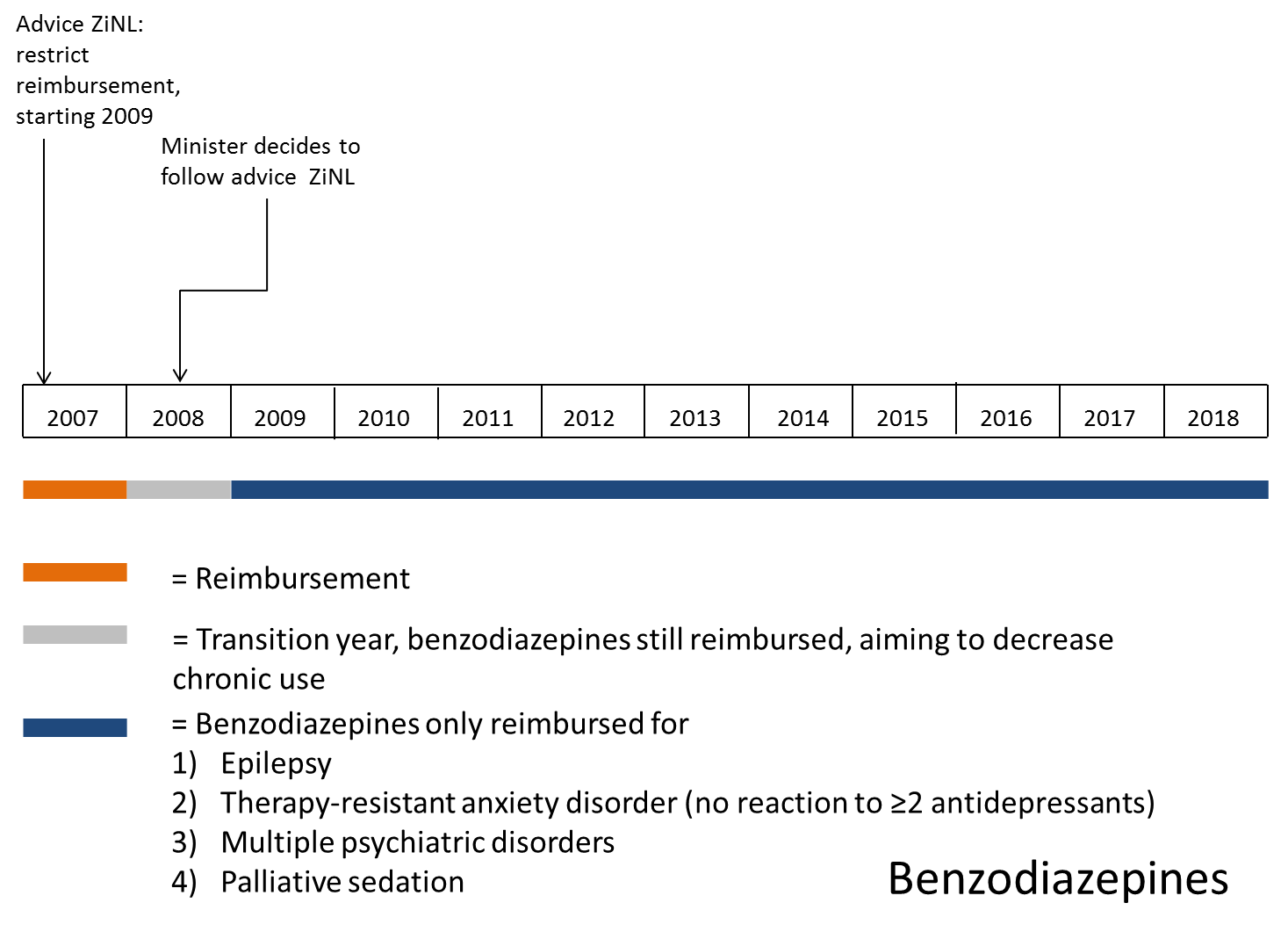


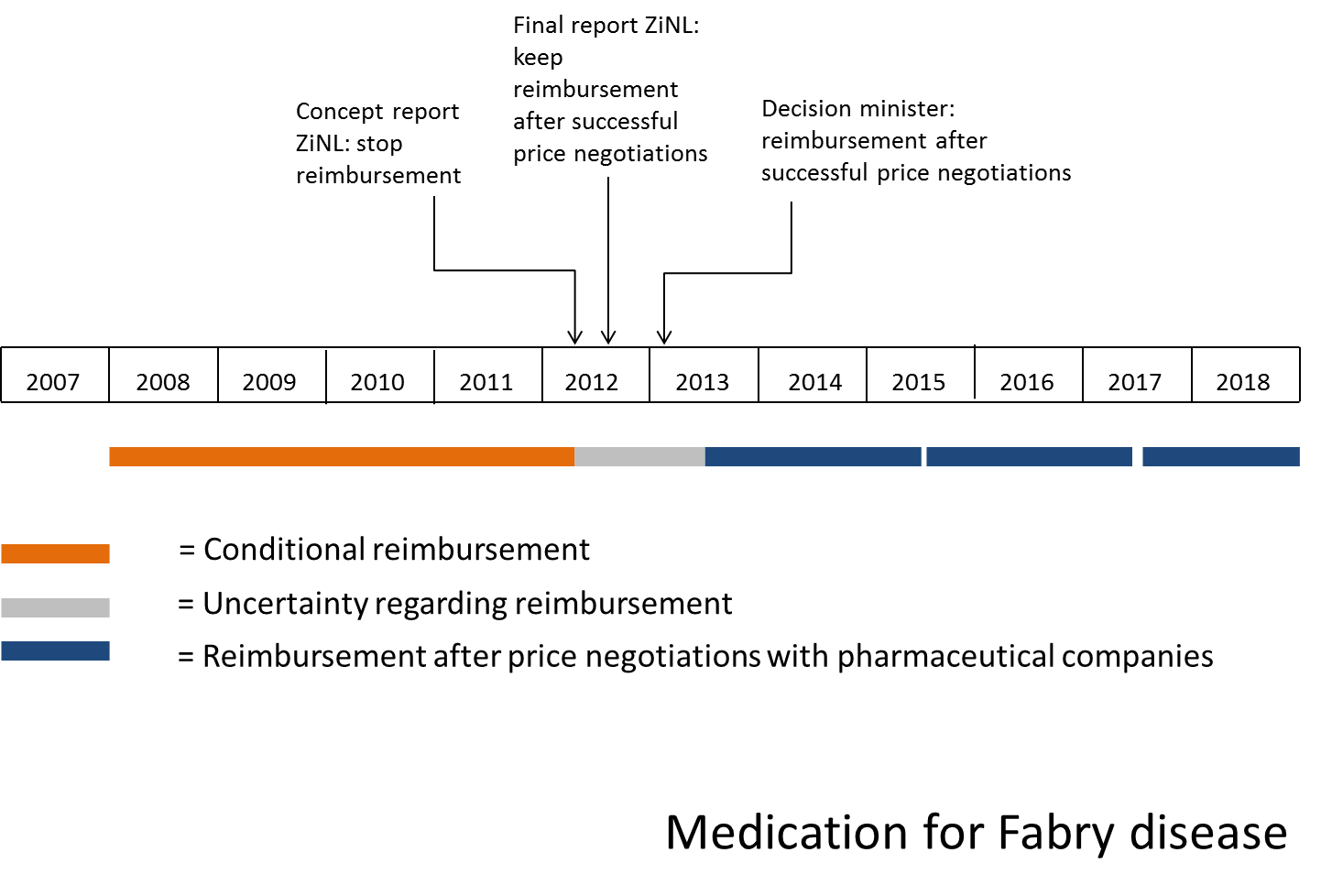


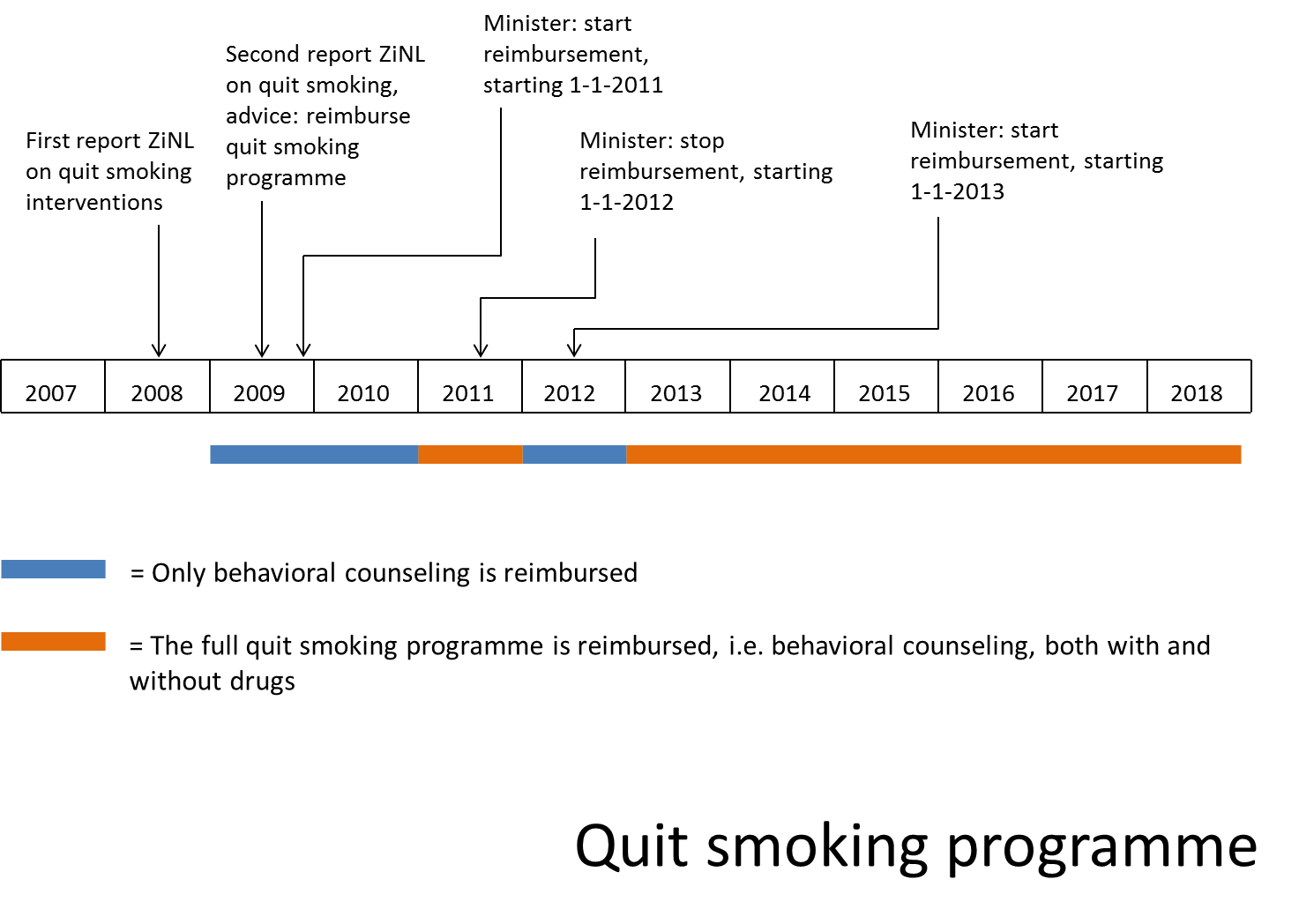


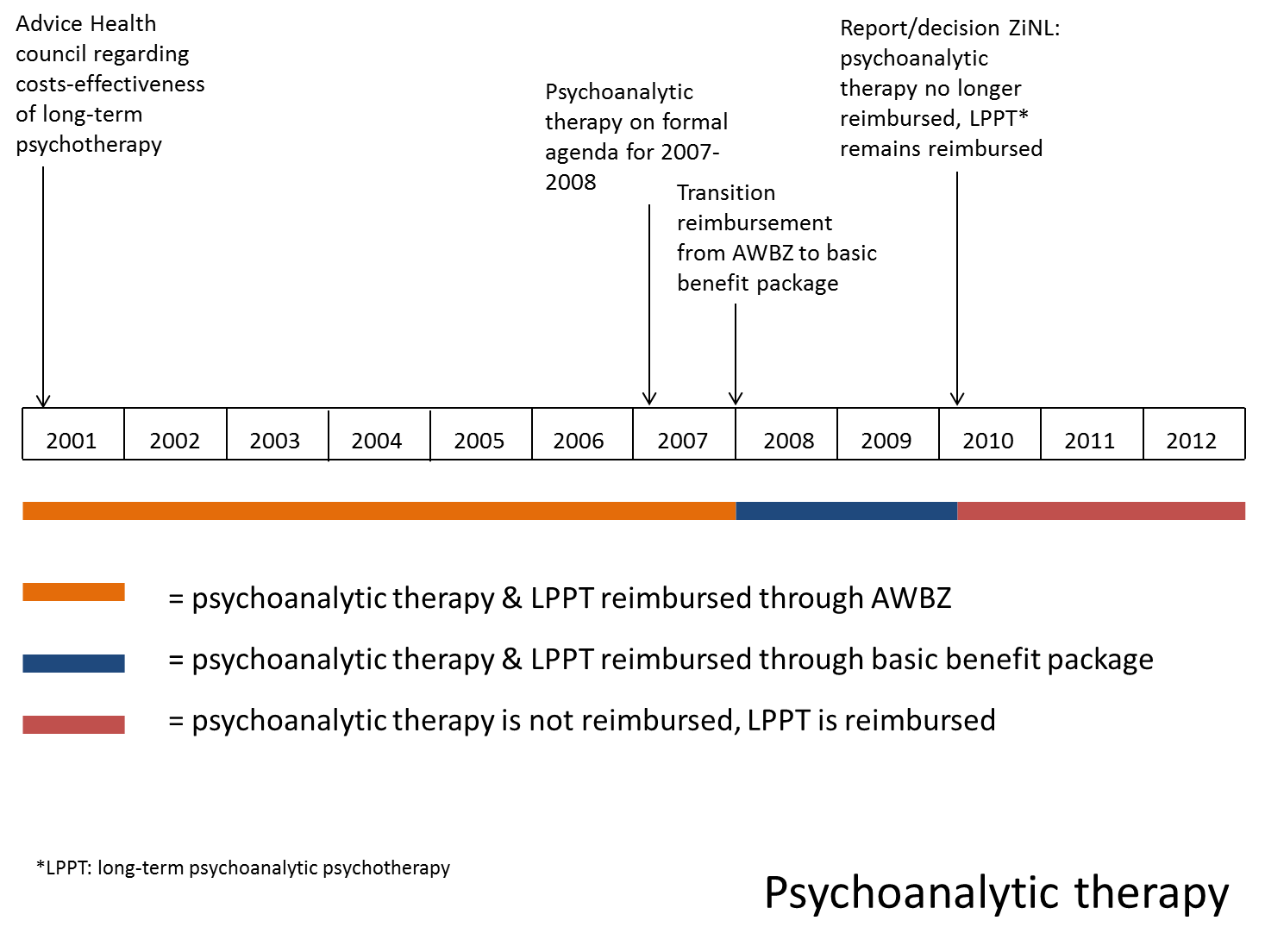


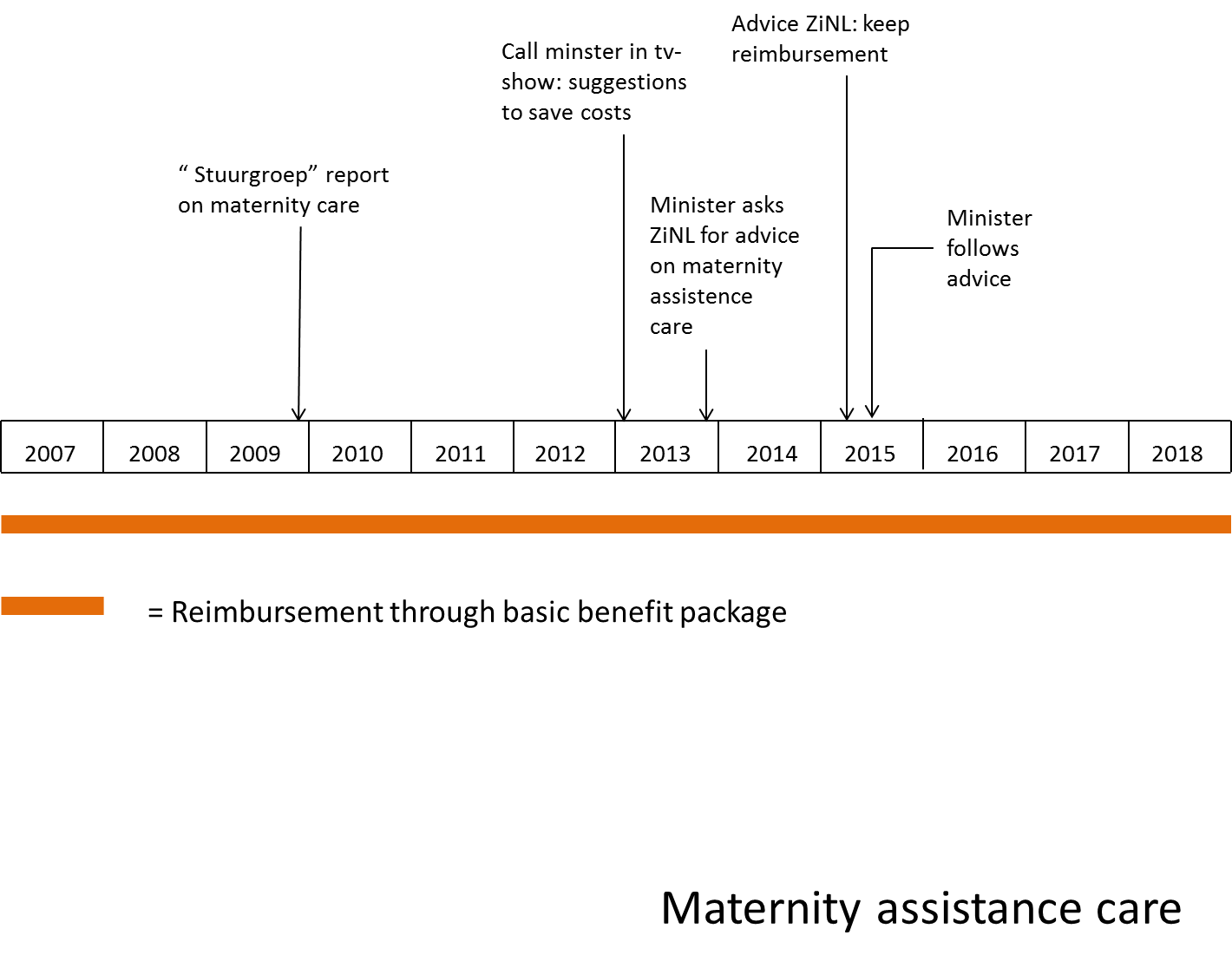


## Additional file F: Themes at case level

| **Themes** | **Cases** | | | | |
| --- | --- | --- | --- | --- | --- |
|  | **A** | **B** | **C** | **D** | **E** |
|  | **Fully disinvested** | **Partially disinvested** | **First disinvested, later reimbursed again** | **Reimbursement maintained** | **Reimbursement maintained** |
| Support for active disinvestment and pressure exerted   - Healthcare providers - Governmental institutions - Patients | Respondents described that there was much societal support for disinvestment, because of the negative view of society on the case. Healthcare providers and patients were against disinvestment, but were not sufficiently able to exert pressure. Respondents described that patients were very vulnerable and, consequently, poorly organized. Furthermore, respondents described that healthcare providers were difficult to mobilize because the small group of healthcare providers was highly divided. According to respondents, governmental institutions were not very open to the input of healthcare providers and patients in this case. | Respondents described that there was broad support for disinvestment among actors, especially because disinvestment was only partial (i.e. several patient groups were excluded from disinvestment) and because of the negative connotation of the case.  They described that, although healthcare providers preferred a more passive approach to disinvestment, they generally supported disinvestment. Respondents described that patients were vulnerable and, consequently, poorly organized. Furthermore, the use of the case intervention is not restricted to a certain patient group. Respondents described that, for these reasons, patients only had a minor role. | Respondents described that political and societal support was very important in this case. Throughout the years, the vision on the case shifted, which affected support for disinvestment.  Furthermore, they described that healthcare providers and interest groups had a large role in reversing disinvestment, because policy makers needed the support from them for other policies as well. Respondents described that, initially, healthcare providers did not exert much pressure against disinvestment. However, after disinvestment took place, healthcare providers exerted much pressure to reverse disinvestment, among others because of a shifted vision on the case.  Respondents described that patients did not have a role in this case because they felt that it would not be considered societally acceptable for them to take any action. | Respondents described that there was broad support for reimbursement, which was driven by the framing of the case in the media. This broad support was essential in maintaining reimbursement.  Respondents described that healthcare providers were very successful in exerting pressure in this case. They were in constant interaction with ZiNL, who carefully considered their input. Furthermore, respondents described that healthcare providers helped patients to exert pressure. In earlier phases of the disinvestment process, patients had a limited role. But later on, when there was media attention, patients could play a somewhat larger role. | Respondents described that some citizens and civil servants were against reimbursement of this case. These people contributed to agenda-setting. However, according to respondents, as other actors were involved in the later stages of the disinvestment process, there was broad support for maintaining reimbursement in these stages. They described that this contributed to reimbursement being maintained. Respondents described that healthcare providers cooperated with each other in exerting pressure. Furthermore, ZiNL was very much open to the input of healthcare providers, with the aim of knowledge sharing. Respondents suggested that these aspects may have facilitated reimbursement being maintained. According to respondents, patient groups were not very visible in the disinvestment process. |
| Compassion for current users | In this case, it was decided to stop reimbursement only for new patients. Current patients could finish their treatment. Respondents described that this was important because actors were reluctant to disrupt treatment. Therefore, they implied that the possibility to stop reimbursement only for new patients substantially contributed to the support for disinvestment. | In this case, several exceptions to disinvestment were made. Respondents described that actors felt that patient groups that really benefited from the case intervention, could still receive reimbursement for this intervention. This facilitated the support for disinvestment for the patient groups who benefitted to a lesser extent from the case intervention. | NA^1^ | There was much variation in effectiveness between patient groups. However, respondents described that, in this case, it was not possible to discern patients who would benefit from the case intervention from patients who would not benefit from this intervention, implying that no exceptions could be made. Respondents described that actors did not want to stop reimbursement for all patients because this would disadvantage patients who could benefit from the case intervention. This contributed to disinvestment being maintained for all patients. | It was not possible to discern patient groups who were more in need for the case intervention from patient groups that were less in need for this intervention, implying that no exceptions could be made. Respondents described that actors were reluctant to stop reimbursement for all patients because this would disadvantage patients who were in greatest need for the case intervention. According to respondent, this contributed to reimbursement being maintained for all patients. |
| Role in health insurance system | According to respondents, as the disinvestment decision was only substantiated by the lack of evidence of effectiveness of the case intervention, the role of the Ministry was limited because they consider decisions based on just effectiveness to be beyond their role (i.e. ZiNL is in charge when it only concerns effectiveness). | Respondents described that healthcare providers did not consider it to be their role to exert pressure. For this reason they tended to stick to comments with regards medical aspects and stay away from exerting pressure. Furthermore, respondents described that health insurers only considered implementation (of disinvestment decisions) as part of their role. For this reason, they were not involved in policy development and decision-making.  Moreover, the ministry considered policy development beyond their role. Therefore, they tried to refrain from involvement in policy-development. | Respondents described that although health insurers supported disinvestment, they did not exert pressure because they did not consider involvement in reimbursement decisions to be their role.  Furthermore, ZiNL also played a limited role, according to respondents, as the decision was mainly political, which ZiNL considers to be the role of the Minister/Ministry. | Respondents described that health insurers felt that involvement in reimbursement decisions does not fit their role. Respondents suggested that this may explain the lack of action undertaken by health insurers. Furthermore, respondents described that the Ministry and ZiNL felt that their role was sequential. First, ZiNL was in the lead. Subsequently, the Ministry/Minister was in the lead. Respondents described that the governmental institutions tended to stick to these roles. | Health insurers described that, although they normally would abstain from being involved in disinvestment processes, in this case, they did exert some pressure because disinvestment would affect provider payment, which they do consider to be part of their role. |
| Financial interest in disinvestment | NA^1^ | Respondents described that because the case intervention was no longer patented, manufacturers did not exert any pressure against disinvestment. | Respondents suggested that, because health insurers would not have much financial benefit from reimbursement (i.e. the health benefits of the case intervention), they were not for reimbursement, although they were also not strongly against reimbursement. | Respondents described that health insurers did not have any financial risk in this case because of the policy rules that were in place. According to respondents, this may have contributed to the lack of action undertaken by health insurers. Respondents also described that manufacturers had strong financial interests in the case. For this reason, manufacturers have tried to exert pressure. | NA^1^ |
| Role of formal package criteria: |  |  |  |  |  |
| - Effectiveness | According to respondents, lack of evidence of effectiveness from comparative studies was the main reason for disinvestment. Patient experiences of effectiveness did not play a role. | According to respondents, lack of effectiveness, combined with many side-effects was the main reason for disinvestment.  Respondents described that effectiveness was also the main reason to make exceptions to disinvestment. | Respondents described that effectiveness only played a role in reversing disinvestment. | According to respondents, lack of evidence of effectiveness combined with limited effectiveness (and very high costs) were the main reasons to consider disinvestment. However, respondents described that lack of evidence of effectiveness was merely an indicator of the need for additional research, not a reason for disinvestment. | Respondents described that there was a lack of scientific evidence of effectiveness. However, there was broad consensus that case stories and qualitative data was sufficient to determine adequacy and soundness of the case intervention. For this reason, effectiveness does not appear to have played a large role. |
| - Cost-effectiveness | Respondents described that the high costs of the case intervention per patient was important. Apart from this, cost-effectiveness did not play a role. | This consideration did not play a role in this case. | This consideration did not play a role in this case. | According to respondents, lack of evidence of effectiveness combined with limited effectiveness and high costs, resulting in a very unfavorable cost-effectiveness ratio, were the main reasons to consider disinvestment. However, respondents described that there was a lack of societal support for a role of cost-effectiveness in reimbursement decisions. Therefore, the role of cost-effectiveness was limited.  Despite this, respondents considered price negotiations essential for reimbursement to be maintained. | This consideration did not play a role in this case. |
| - Necessity^2^ | These considerations did not play a role in this case. | Respondents described that whether it would be feasible for patients to pay the case interventions themselves, was an important reason for disinvestment and played a major role in determining the exceptions (i.e. patient groups for whom reimbursement would be maintained). Furthermore, whether treatment was considered medically necessary was also very important in determining the exceptions. | According to respondents, whether patients can pay for the case intervention themselves, and individual and societal responsibility to pay for the case intervention, were the main arguments in the discussion on reimbursement. Other necessity aspects that played a smaller role were the disease burden and the large number of patients. | Respondents described that medical necessity of the case interventions was evident, because of disease severity, lack of alternative treatment and the small number of patients. This contributed to maintaining reimbursement. | Respondents described that whether patients can afford to pay for the case intervention themselves and individual and societal responsibility to pay for the case intervention, played a large role in policy development and decision-making in this case. |
| - Feasibility^3^ | These considerations did not play a role in this case. | Respondents described that the large budget-impact played a role in this case. Furthermore, they described that the administrative burden of making exceptions to disinvestment played a major role to determine the exceptions made (i.e. patient groups for whom reimbursement would be maintained). | Respondents described that budget-impact only played a role in reversing disinvestment. Other feasibility considerations did not play a role in this case. | These considerations did not play a role in this case. | According to respondents, feasibility considerations, such as budget impact, and organization and registration of care have also played a role. |
| - Other^4^ |  |  |  |  | Respondents described that solidarity considerations were important in agenda-setting. |
| ^1^NA: not applicable. Nothing with regards to this theme was addressed in the interviews.  ^2^Necessity covers both the medical necessity of an intervention and the necessity to insure an intervention. Medical necessity covers aspects such as disease burden, rule of rescue and whether there is an alternative treatment available. Necessity to insure covers aspects such as ‘who is responsible for paying for the intervention’ and ‘can patients afford to pay for the intervention themselves’. See for more information the paper of Kleinhout-Vliek et al. (Kleinhout-Vliek et al., 2017)  ^3^ Feasibility covers aspects such as budget-impact (i.e. is paying feasible?), indication and administration (e.g. administrative burden, feasibility of regulations), and organization of care (e.g. can the intervention be (de-)implemented in the current organization of care?).  ^4^This covers considerations that played a major role, but are beyond the formal package criteria. If no other considerations played a role, this cell is left blank. | | | | | |

## Additional file G: Consolidated criteria for reporting qualitative studies (COREQ): 32-item checklist

| **No .** | **Item .** | **Guide questions/description .** | **Response** |
| --- | --- | --- | --- |
| **Domain 1: Research team and reflexivity** | | | |
| Personal Characteristics | | | |
| 1. | Interviewer/facilitator | Which author/s conducted the interview or focus group? | A.H. Rotteveel conducted all interviews. In four interviews, G.A. de Wit or N.J.E. van Vooren (not a co-author) joined as well. See page 9. |
| 2. | Credentials | What were the researcher's credentials? *E.g. PhD, MD* | A.H. Rotteveel, MSc  G.A. de Wit, PhD  N.J.E. van Vooren, |
| 3. | Occupation | What was their occupation at the time of the study? | A.H. Rotteveel: PhD candidate  G.A. de Wit: senior health economist  N.J.E. van Vooren: junior researcher |
| 4. | Gender | Was the researcher male or female? | All interviewers are female. |
| 5. | Experience and training | What experience or training did the researcher have? | A.H. Rotteveel: Research master health sciences with a course on qualitative research. Experience with interviews, focus groups, and questionnaire testing in previous research projects.  G.A. de Wit: Master in health sciences. Experience with supervising interviewstudies.  N.J.E. van Vooren: Master in health and society. Ample experience with qualitative studies. |
| Relationship with participants | | | |
| 6. | Relationship established | Was a relationship established prior to study commencement? | No |
| 7. | Participant knowledge of the interviewer | What did the participants know about the researcher? e*.g. personal goals, reasons for doing the research* | The participants knew that the researcher was a PhD candidate and that research was financed using an internal research grant. They knew the study was not ordered by the Ministry or ZiNL. |
| 8. | Interviewer characteristics | What characteristics were reported about the interviewer/facilitator? e.g. *Bias, assumptions, reasons and interests in the research topic* | None |
| **Domain 2: study design** | | | |
| Theoretical framework | | | |
| 9. | Methodological orientation and Theory | What methodological orientation was stated to underpin the study? *e.g. grounded theory, discourse analysis, ethnography, phenomenology, content analysis* | Content analysis, see page 7, paragraph 2.4 |
| Participant selection | | | |
| 10. | Sampling | How were participants selected? *e.g. purposive, convenience, consecutive, snowball* | Purposive sampling, based on policy documents, media coverage and previous interviews, see page 6, paragraph 2.4 |
| 11. | Method of approach | How were participants approached? e*.g. face-to-face, telephone, mail, email* | Respondents were approached through e-mail, see page 6, paragraph 2.3 |
| 12. | Sample size | How many participants were in the study? | 37, see page 8 and 9, paragraph 3 |
| 13. | Non-participation | How many people refused to participate or dropped out? Reasons? | Of the approached actors, 3 did not reply, 2 replied that they were not willing to participate and 6 were organizations that were not able to find out which employees were involved in the case. The remaining 37 actors agreed to participate. None of these dropped out at later stages. |
| Setting | | | |
| 14. | Setting of data collection | Where was the data collected? e*.g. home, clinic, workplace* | Interviews were held at the location best suitable for the participant. Locations chosen by participants were: at workplace (of participant), at home (of participant), at external location (rented meeting room) and at the RIVM (workplace interviewer). |
| 15. | Presence of non-participants | Was anyone else present besides the participants and researchers? | No |
| 16. | Description of sample | What are the important characteristics of the sample? *e.g. demographic data, date* | See table 1 |
| Data collection | | | |
| 17. | Interview guide | Were questions, prompts, guides provided by the authors? Was it pilot tested? | The interview guide has been included as additional file C. |
| 18. | Repeat interviews | Were repeat interviews carried out? If yes, how many? | No |
| 19. | Audio/visual recording | Did the research use audio or visual recording to collect the data? | Audio recording, see page 7, paragraph 2.3 |
| 20. | Field notes | Were field notes made during and/or after the interview or focus group? | Fieldnotes were only taken as back-up for the audio-recording. They were only used if audio recording would fail. |
| 21. | Duration | What was the duration of the interviews or focus group? | De average duration of the audio recording was 53 minutes (see page 8, paragraph 3) with a range of 14 to 99 minutes. The variation in interviews duration was caused by different levels of involvement of respondents and because some respondents were interviewed over multiple cases (for each case, audio was recorded separately).. |
| 22. | Data saturation | Was data saturation discussed? | Yes, see page 9 |
| 23. | Transcripts returned | Were transcripts returned to participants for comment and/or correction? | Yes |
| **Domain 3: analysis and findings** | | | |
| Data analysis | | | |
| 24. | Number of data coders | How many data coders coded the data? | Two: AR and JvdR. See page 8, paragraph 2.4 |
| 25. | Description of the coding tree | Did authors provide a description of the coding tree? | Yes, see additional file D |
| 26. | Derivation of themes | Were themes identified in advance or derived from the data? | Both. A combination of deductive and inductive coding was used, see page 7, paragraph 2.4 and additional file D. |
| 27. | Software | What software, if applicable, was used to manage the data? | MAXQDA 2019, see page 8, paragraph 2.4 |
| 28. | Participant checking | Did participants provide feedback on the findings? | Yes, participants had the opportunity to check the usage of their quotes in the paper (see page 7, paragraph 2.3.1). Furthermore, they will be informed on the results after publication of this paper. |
| Reporting | | | |
| 29. | Quotations presented | Were participant quotations presented to illustrate the themes / findings? Was each quotation identified? e*.g. participant number* | Yes, yes. |
| 30. | Data and findings consistent | Was there consistency between the data presented and the findings? | Yes. |
| 31. | Clarity of major themes | Were major themes clearly presented in the findings? | Yes. |
| 32. | Clarity of minor themes | Is there a description of diverse cases or discussion of minor themes? | Yes. |

1. In the Netherlands, many women with low-risk pregnancies give birth at home. Women that do give birth at the hospital or a birth centre are often discharged after a few hours to recover at home as well. Only women/newborns with severe complications are admitted to the hospital for a longer time. [↑](#footnote-ref-1)
2. The exact questions that were asked were formulated during the interviews, based on the responses of the respondent. However, interviewers made sure that all topics were covered during the interviews. [↑](#footnote-ref-2)
